# Entropy gain due to water release upon ligand binding

**DOI:** 10.1186/1758-2946-6-S1-P35

**Published:** 2014-03-11

**Authors:** Mazen Ahmad, Olga Kalinina, Thomas Lengauer

**Affiliations:** 1 Max-Planck-Institut für Informatik , Saarbrücken, Campus E1-4, 66123, Germany

## 

Experimental thermodynamic data of the ligand-receptor association showed that the entropy changes upon binding are positive and large enough to be important driving forces of the binding process for a considerable number of ligand-receptor complexes [1]. The expected source behind such an entropy increase is the release of the water molecules from the binding pocket and from around the ligand to get more freedom in the bulk phase. However, this important source of entropy is usually ignored due to the lake of a method to compute it. Therefore, we developed a method to compute the entropic gain due to water release upon ligand binding.

Water molecules close to a charged residue are restricted from free rotation due to the interaction between their dipole moments and the electric field. This restriction result in a decrease in their entropy. The loss of entropy can be gained again when the water molecules displaced from area that under the electric field upon the binding process. To compute the loss of entropy due to the electric field, we developed a method based on the knowledge of the electric field which affects the rotation of the water molecules using the continuum electrostatic methods. This provides a possibility for the calculation of the entropic loss from the partition function of the water molecules under an electric field. For monovalent ions, the computed loss in entropy upon solvation correlates well with experimentally measured one.

The loss of entropy of water close to hydrophobic molecules (Hydrophobic entropy) cannot be explained by the effect of the electric field from the molecule because the magnitude of the electric field is not strong enough to restrict the mobility of the water molecules. However, the loss in entropy results from the asymmetric interactions of the water molecules with the hydrophobic molecules and the neighbour water molecules. To compute the hydrophobic entropy we developed an empirical method which is derived from a linear relation between the hydration entropy of small hydrophobic molecules and the hydrophobicity of the water molecules around the surface of the small molecule. A training data set of 142 small molecules shows a significant linear relation between the hydrophobic entropy and the computed hydrophobicity.
